# Antioxidants for Preventing Preeclampsia: A Systematic Review

**DOI:** 10.1100/2012/243476

**Published:** 2012-04-19

**Authors:** Adriana Magalhaes Ribeiro Salles, Tais Freire Galvao, Marcus Tolentino Silva, Lucilia Casulari Domingues Motta, Mauricio Gomes Pereira

**Affiliations:** University of Brasilia, Faculty of Medicine, Asa Norte, 70910-900 Brasilia, DF, Brazil

## Abstract

*Objective*. To investigate the efficacy of antioxidants for preventing preeclampsia and other maternal and fetal complications among pregnant women with low, moderate, or high risk of preeclampsia. *Methods*. We searched MEDLINE, Embase, CENTRAL, mRCT, and other databases, with no language or publication restrictions. Two independent reviewers selected randomized controlled trials that evaluated the use of antioxidants versus placebo and extracted the relevant data. Relative risks (RRs) and 95% confidence intervals (95% CIs) were calculated. The data were compiled through the random effects model. *Main Results*. Fifteen studies were included (21,012 women and 21,647 fetuses). No statistically significant difference was found between women who received antioxidant treatment and women who received placebo for preeclampsia (RR  = 0.92; 95% CI: 0.82–1.04), severe preeclampsia (RR  = 1.03; 95% CI: 0.87–1.22), preterm birth (RR  = 1.03; 95% CI: 0.94–1.14), and small for gestational age <10th centile (RR  = 0.92; 95% CI: 0.80–1.05). Side effects were numerically more frequent in the antioxidants group compared to placebo, but without significant statistical difference (RR  = 1.24; 95% CI: 0.85–1.80). *Conclusions*. The available evidence reviewed does not support the use of antioxidants during pregnancy for the prevention of preeclampsia and other outcomes.

## 1. Introduction

Hypertensive disorders during pregnancy are the most common cause of maternal death in Latin America and the Caribbean accounting for 25.7% of all maternal deaths; in developed countries, the corresponding proportion is lower, yet still significant: 16.1% [1]. Reducing maternal mortality by three quarters by 2015 is one objective of the Millennium Development Goals (MDGs) of the United Nations Development Programme [2].

Although several hypotheses have been proposed, the causes of preeclampsia remain unclear. There is a relationship between placental insufficiency and the pathophysiology of preeclampsia. Placental oxidative stress plays an important role in the manifestations of preeclampsia [3]. Oxidative stress and lipid peroxidation accompany complications such as the occurrence of endothelial cell dysfunction in the blood vessels in women with preeclampsia and other hypertensive disorders [4, 5]. Antioxidants might be important for the prevention of lipid peroxidation and, hypothetically, for the prevention of preeclampsia [3]; however, the evidence of antioxidants efficacy for preventing preeclampsia has not been confirmed yet [6, 7].

The objective of this study was to systematically review randomized trials of low-, moderate-, or high-risk women treated with antioxidants to prevent preeclampsia and other maternal or fetal complications.

## 2. Methods

### 2.1. Studies Eligibility Criteria

We considered eligible randomized controlled trials that enrolled women with low, moderate, or high risk of preeclampsia that used antioxidants compared to placebo or no antioxidants, to assess antioxidants effect in preeclampsia. If unpublished reports were detected, we contacted studies' authors to obtain the data of interest.

High risk of preeclampsia was defined as previous severe preeclampsia, diabetes, chronic hypertension, renal disease, or autoimmune disease. Moderate/low risk was defined as women who did not meet the criteria for high risk or have first pregnancy, a mild rise in blood pressure and no proteinuria, positive roll-over test, abnormal uterine artery Doppler scan, multiple pregnancy, a family history of preeclampsia, maternal age less than 20, and known thrombophilia. When the risk was unclear or unspecified, women were classified as moderate/low risk [6].

### 2.2. Sources and Search Strategies

Literature search was performed with no language restrictions and no limits on publication date. The research was done on MEDLINE, Embase, Cochrane Central Register of Controlled Trials (CENTRAL), *meta*Register of Controlled Trials (mRCT), Centre for Reviews and Dissemination (CDR), ISI of Web Science, Scopus, Latin American and Caribbean Center on Health Sciences Information (LILACS), and Scientific Electronic Library Online (SciELO) databases. References from relevant studies were also researched to identify potentially eligible studies. To identify the grey literature, ProQuest Dissertation and Theses and Brazilian theses registration databases were searched, as well as websites of gynecology and obstetrics associations. Last literature search was performed in October 2011.

Search strategy used in MEDLINE (via PubMed) was ((“pre-eclampsia” [mesh] or “pre-eclampsia” [tiab] or preeclampsia [tiab] and “pregnancy complications” [mesh] or “pregnancy” [mesh] or “pregnancy” [tiab]) and (“antioxidants” [tiab] or “antioxidants” [mesh] or “antioxidants” [pharmacological action] or “antioxidant” [tiab]) or “ascorbic acid” [mesh] or “ascorbic-acid” [tiab] or “ascorbic acid” [tiab] or “vitamin c” [tiab] or “vitamin e” [mesh] or “vitamin-e” [tiab] or “alpha-tocopherol” [mesh] or alphatocopherol [tiab] or “beta carotene” [mesh] or “beta-carotene” [tiab] or “selenium” [mesh] or selenium [tiab] or “glutathione peroxidase” [mesh] or “glutathione peroxidase” [tiab] or “superoxide dismutase” [mesh] or “superoxide dismutase” [tiab] or “catalase” [mesh] or “catalase” [tiab]) and (therapy/narrow [filter]). We adapted this strategy for searching on the other databases.

### 2.3. Studies Selection

Two reviewers (LDCM, AMRS) selected the articles in an independent, unblinded manner, by reading the studies' titles and abstracts. Cases of disagreement were resolved in consensus meetings.

### 2.4. Data Extraction

Two reviewers (LDCM, AMRS) extracted data independently on a purpose-built electronic form. In the event of disagreement, the decision was taken by reaching a consensus or by an independent reviewer (TFG). We extracted from studies the year, country, funding source, type of study, sample size, group allocation, population characteristic, intervention, primary outcomes and secondary outcomes. We contacted the corresponding author of included studies if any data were not available in the paper.

### 2.5. Quality and Risk of Bias Assessments

This assessment was made independently by two reviewers (LDCM, AMRS), using the Cochrane Collaboration method [8]. We evaluated the following items: random sequence generation, allocation concealment, blinding of participants and personnel, blinding of outcome assessment, incomplete outcome data, selective reporting, and other bias (such as an insensitive instrument used to measure outcomes, selective reporting of subgroups and baseline imbalance in factors that are strongly related to outcome).

Sensitivity analysis of the global effect was conducted to verify the impact of studies of lower quality on the primary outcome: such studies were excluded from the analysis and the results were compared to the full analysis.

Funnel plot asymmetry was assessed and grey literature search was included to minimize the risk of publication bias [8]. We also calculated Peters' test for small-study effects [9] and Harbord's modified test for small-study effects [10] to objectively detect publication bias.

Excluded studies due to full text not being available were included in primary outcome meta-analysis to assess their impact on global effect, publication bias, and heterogeneity (sensitivity analysis).

### 2.6. Outcomes

The primary outcome measured was the relative risk (RR) of preeclampsia. Secondary outcomes were severe preeclampsia (including HELLP syndrome—hemolysis, elevated liver enzymes, and low platelet count, and imminent eclampsia), preterm birth (less than 37 completed weeks of pregnancy), small for gestational age infants (defined as smaller than the third, smaller than the fifth, and smaller than the tenth percentile), and baby death (miscarriage, stillbirth, neonatal, and infant death). The incidence of side effects was also verified.

### 2.7. Statistical Analysis

Statistical analysis was based on the calculated relative risks and their respective 95% confidence intervals (95% CI) for each study reviewed. The data from all the studies were compiled based on the Mantel-Haenszel test, through the random effects model. Analysis and graphs were obtained by using Review Manager 5 (version 5.1.6) and STATA (version 10.1). The chi-squared tests (*P* ≤ 0.10), *I* ^²^, and Tau² were calculated to assess heterogeneity among the studies. Studies with moderate or substantial heterogeneity were explored to identify possible causes for inconsistency [8]. If absolute values were absent, we calculated them from relative results available on the reports.

## 3. Results

**Figure 1:**
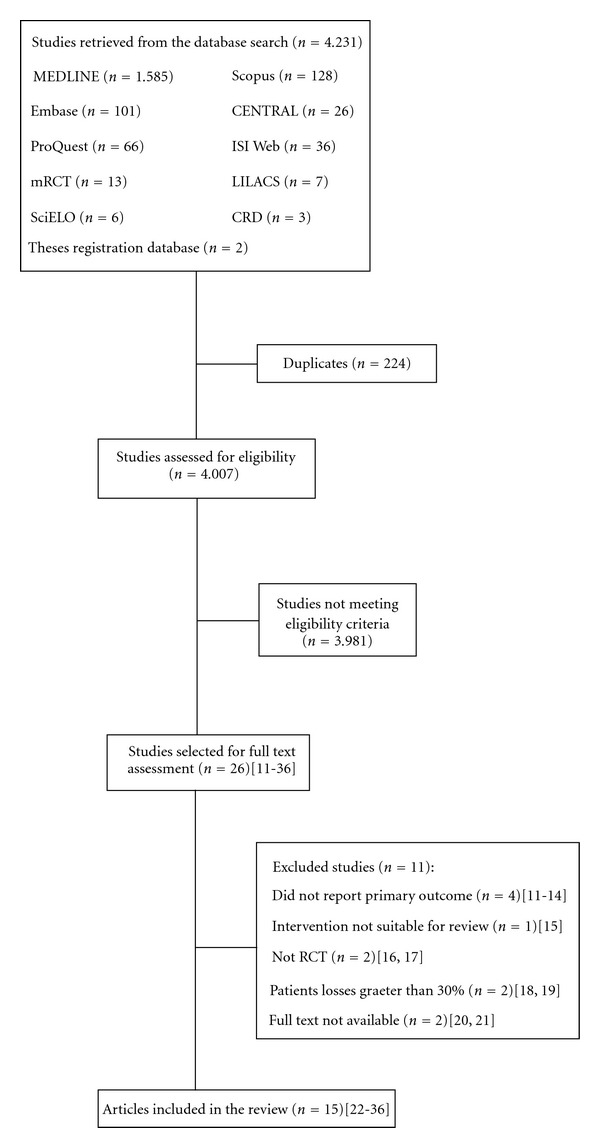
Flow chart of the search, selection, and inclusion of studies.

A total of 4,231 studies were retrieved and 15 were included in our analysis (Figure 1). All studies were randomized placebo-controlled trials that assessed including 21,012 women and 21,647 fetuses. The main characteristics of included studies are shown in Table 1.

### 3.1. Quality and Risk of Bias Assessments

**Figure 2:**
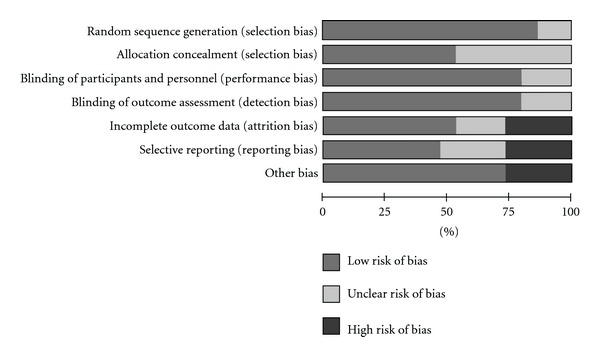
Quality assessment and risk of bias.

Quality assessment result is shown in Figure 2. Three articles satisfied all quality assessment criteria [8, 27, 28]. In all the items analyzed, at least ~50% of the articles presented a low risk of bias. Roughly 30% of the articles presented a high risk of bias on the items: incomplete outcome data [29, 31, 34], selective reporting [26, 31, 34, 35], and other bias [23, 24, 33], that included insensitive instrument used to measure outcomes and deviation from the study protocol.

**Figure 3:**
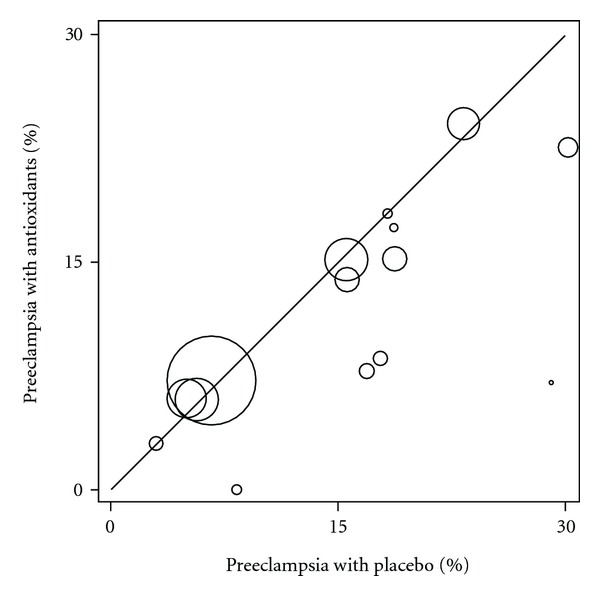
Preeclampsia incidence proportion with antioxidants and placebo groups.

Inspection of the funnel plots for preeclampsia medical outcome (data not presented) revealed asymmetric results, indicating a risk of publication bias. This risk was found to be statistically significant by Peters' test (*P* = 0.005) and Harbord's modified test (*P* = 0.004) for small-study effects. We found a higher number of smaller studies that favored antioxidants, suggesting that similar studies that favored control group were not published. In Figure 3, a L'Abbé plot, each trial is represented by a circle whose diameter is proportional to the population size. Larger studies are located along the no difference line (RR = 1), while smaller studies show worse results with placebo group.

Several studies did not report any side effects. This could be considered selective reporting bias. It was not possible to obtain the protocols of these randomized controlled trials to check whether reporting this outcome was planned. The authors may not have considered the incidence of side effects as a relevant outcome and thus refrained from collecting such data. Due to these uncertainties, the omission of side effects on studies results was not considered as selective reporting.

Two studies did not publish their data in full text, just as conference abstracts [20, 21], what prevented us to perform their quality assessment and the studies were excluded from analysis (Figure 1). Sensitivity analysis was done to assess such exclusion impact on publication bias. By including these studies in the paper, the asymmetry of the funnel plot increased, as well as heterogeneity of preeclampsia outcome (Chi² 28.88, df 15 (*P* = 0.02); *I* ^²^ 45%). Furthermore, the risk of preeclampsia was numerically lower (RR = 0.90; 95% CI: 0.78–1.03).

### 3.2. Outcomes

**Figure 4:**
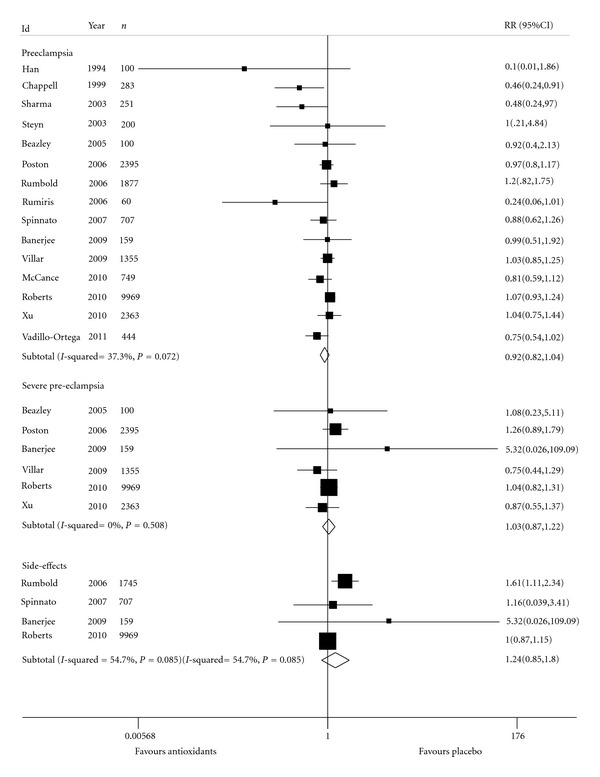
Maternal outcomes. Comparison: antioxidants versus placebo.

There was no statistically significant difference for preeclampsia incidence when comparing women who received antioxidants and the placebo group (*n* = 21, 012; RR = 0.92; 95% CI: 0.82–1.04; Figure 4). Only two studies revealed a significant result of reduced occurrence preeclampsia in the group of women who used antioxidant compared to the placebo group [23, 24]. No difference was noted in severe preeclampsia (*n* = 16, 341; RR = 1.03; 95% CI: 0.87–1.22; Figure 4).

Preterm birth, small for gestational age <3rd centile, small for gestational age <5th centile, small for gestational age <10th centile, miscarriage, and neonatal death were also found not to be statistically significant (Table 2).

The estimates of preterm birth and small for gestational age infants were heterogeneous. Analysis of this heterogeneity causes showed that it is probably due to differences in population [32, 33] and interventions [31, 35].

Women who took antioxidants presented an increased number of side effects compared to women who took placebo but no statistically significant difference between the groups analyzed was identified (*n* = 12, 580; RR = 1.24; 95% CI: 0.85–1.80). Reported effects were abdominal pain at the end of pregnancy [28, 30]: itching, eczema, vomiting, diarrhea, headache, constipation, malaise, decreased vision [30], skin rash, and chest pain [31]. One study reported nausea and vomiting as side effects [34]. To avoid duplication of participants, we only included nausea data in the meta-analysis. Another trial reported no occurrence of side effects but only assessed changes in blood and urine analysis or in liver or renal function [34]. The polled estimate of side effects showed to be heterogeneous. Exploring this heterogeneity we noticed clinical and methodological differences across studies.

The sensitivity analysis of the primary outcome considering only studies that fulfilled all quality criteria (Poston 2006 [27], Rumbold et al. 2006 [28], and Villar et al. 2009 [32]) revealed a nonsignificant increased risk of preeclampsia (*n* = 5, 627; RR = 1.02; 95% CI: 0.90–1.16; heterogeneity: Chi² *P* value = 0.60; *I* ^²^ = 0%).

## 4. Discussion

Antioxidants efficacy for preventing preeclampsia was not observed from included studies and results from these studies are prone to have publication bias, what reduces the confidence of the findings. Only two isolated studies showed a significant reduction of preeclampsia in women treated with antioxidants compared to placebo, but important differences were present, mainly on interventions. Efficacy was also not detected for other outcomes assessed. The large number of women randomly investigated leads us to believe that additional studies would probably not alter this result.

The sensitivity analysis, when including only studies that met all quality criteria, revealed a nonsignificant increased risk of preeclampsia, while the analysis including all studies reduced the risk, also without statistically significant difference between antioxidants and placebo.

Heterogeneity across studies was not significant for the outcomes preeclampsia, severe preeclampsia, or baby death. Moderate heterogeneity was found for small for gestational age, preterm birth and side effects. This may have been due to clinical and methodological differences identified in some of the studies. However, due to their large sample size, heterogeneity tests can identify small statistical heterogeneous portions that may not be clinically important [8].

### 4.1. Previous Systematic Reviews

We found seven systematic reviews that analyzed the efficacy of antioxidants in the prevention of preeclampsia and other maternal and fetal outcomes [6, 7, 37–41]. Five reviews showed no statically significant difference for the outcomes analyzed [6, 7, 39–41]. Of these, four tested the efficacy of the combination of vitamins C and E [7, 39–41]. One review assessed the efficacy of any antioxidant and found no statically significant difference in the assessed outcomes, except for the side effects [6].

Another review assessed only vitamin C as antioxidant and showed a higher risk of preterm birth in women who took vitamin C compared to placebo group, but a lower risk of preeclampsia in those treated with antioxidant [37]. Other outcomes were not statistically significant. Meanwhile, the review that analyzed only vitamin E also found a lower risk of preeclampsia among the group who took the vitamin versus placebo, with no statically significant difference from other outcomes [38]. Despite these reviews stated to analyze only vitamin C or E effects, other antioxidants were included and the total number of included women was less than 1,000 in both reviews, thus showing small precision.

One study performed subgroup risk analysis for preeclampsia to test the antioxidant effect [39]. No statistically significant differences between analyzed groups were found.

### 4.2. Strengths and Limitations of the Review

This review presents a method in line with the current recommendations for systematic reviews: sensitive search, no restrictions on language or publication date, search for grey literature, paired selection, and data extraction [8, 42]. Such measures are required to avoid biases and reveal transparent and faithful results.

Furthermore, meta-analyses were conducted following the random effects model. The results were subjected to sensitivity analysis and assessment of publication bias and heterogeneity across results. This procedure aimed to identify and explain possible biases.

Although two studies were excluded due to absence of full text, we assessed the impact of their exclusion on the funnel plot asymmetry, heterogeneity, and outcome estimates. It was shown that the inclusion of these studies would not lead to important change in the results.

We intended to test the efficacy of any antioxidants because other systematic reviews had shown nonsignificant results for vitamin C and E for preventing preeclampsia. As most randomized controlled trials only analyzed these two vitamins, such studies influenced the results, rending not sufficient data to test the efficacy of other antioxidants than vitamin C and E.

There are a reasonable number of studies that verify the efficacy of antioxidants for preventing preeclampsia, resulting in a large number of women assessed. The number of included patients in our review was three times larger than the last Cochrane Review that also reviewed the efficacy of any kind of antioxidants [6], what is likely to imply in greater precision in the analysis.

## 5. Conclusion

 Available evidence does not support the use of antioxidants during pregnancy. Their use in pregnancy for the prevention of preeclampsia and other maternal and fetal outcomes should be well balanced, as beneficial effects are not proved.

## Figures and Tables

**Table 1:** Characteristics of the included studies.

Study	Country	Population	Intervention doses		Assessed outcomes
			(mg)	(UI)	
Han and Zhou 1994 [22]	China	100 women with low/moderate risk of preeclampsia. Gestational age at entry not informed	Selenium: 0.1		Preeclampsia, side effects
Chappell 1999 et al. [23]	UK	283 women with low/moderate and high risk of preeclampsia with gestational age between 16 and 22 weeks	Vitamin C: 1000	Vitamin E: 400	Preeclampsia, preterm birth <37 weeks, small for gestational age <10th percentile, baby death, miscarriage
Sharma 2003 et al. [24]	India	251 primigravidae with low/moderate risk of preeclampsia between 16 and 20 weeks of gestation	Lycopene: 4		Preeclampsia, small for gestational age <10th percentile
Steyn 2003 et al. [25]	South Africa	200 women with low/moderate risk of preeclampsia with gestational age under 26 weeks	Vitamin C: 500		Preeclampsia, preterm birth <37 weeks, baby death, miscarriage, neonatal death
Beazley 2005 et al. [26]	USA	109 women with low/moderate and high risk of preeclampsia with gestational age between 14 and 20 weeks	Vitamin C: 1000	Vitamin E: 400	Preeclampsia, severe preeclampsia, preterm birth <37 weeks, small for gestational age <10th percentile
Poston 2006 et al. [27]	UK and Holland	2395 women with low/moderate and high risk of preeclampsia with gestational age between 14 and 21 weeks	Vitamin C: 1000	Vitamin E: 400	Preeclampsia, severe preeclampsia, preterm birth <37 weeks, small for gestational age <5th and <10th percentile, baby death, miscarriage, neonatal death
Rumbold 2006 et al. [28]	Australia	1877 nulliparous women with low/moderate risk of preeclampsia between 14 and 21 weeks of gestation	Vitamin C: 1000	Vitamin E: 400	Preeclampsia, small for gestational age <10th and 3rd percentile, preterm birth <37 weeks, baby death, miscarriage, neonatal death, adverse effects
Rumiris 2006 et al. [29]	Indonesia	60 women with low/moderate and high risk of preeclampsia with gestational age between 8 and 12 weeks	Vitamin B-6: 2.2; vitamin B-12: 0.0022; vitamin C: 200; folic acid: 0.4; n-acetylcysteine: 200; copper: 2; zinc: 15; manganese: 0.5; iron: 30; calcium: 800; selenium: 0.1	Vitamin A: 1000; vitamin E: 400	Preeclampsia, preterm birth <37 weeks, miscarriage
Spinnato 2007 et al. [30]	Brazil	707 women with high risk of preeclampsia between 12 and 19 weeks	Vitamin C: 1000	Vitamin E: 400	Preeclampsia, severe preeclampsia, preterm birth <37 weeks, small for gestational age <10th percentile, baby death, stillborn/miscarriage, neonatal death, adverse effects
Banerjee 2009 et al. [31]	India	159 primigravidae with low/moderate risk of preeclampsia between 12 and 20 weeks of gestation	Lycopene: 2		Preeclampsia, severe preeclampsia, preterm birth, baby death, adverse effects
Villar 2009 et al. [32]	India, Peru, South Africa, Vietnam	1365 women with low/moderate and high risk of preeclampsia with gestational age between 14 and 22 weeks	Vitamin C: 1000	Vitamin E: 400	Preeclampsia, severe preeclampsia, preterm birth <37 weeks, small for gestational age <10th percentile, baby death
McCance 2010 et al. [33]	Ireland, Scotland, England	762 women with high risk of preeclampsia with gestational age between 8 and 22 weeks	Vitamin C: 1000	Vitamin E: 400	Preeclampsia, preterm birth <37 weeks, neonatal death
Roberts 2010 et al. [34]	USA	9969 women with low/moderate risk of preeclampsia with gestational age between 9 and 16/6 weeks	Vitamin C: 1000	Vitamin E: 400	Preeclampsia, severe preeclampsia, preterm birth <37 weeks, small for gestational age <3rd percentile, baby death, miscarriage, neonatal death; side effects
Xu 2010 et al. [35]	Canada and Mexico	2363 women with low/moderate and high risk of preeclampsia with gestational age between 12 and 18 weeks	Vitamin C: 1000	Vitamin E: 400	Preeclampsia, severe preeclampsia, preterm birth <37 weeks, small for gestational age <10th and <5th percentile, baby death, miscarriage/stillbirth, neonatal death.
Vadillo-Ortega 2011 et al. [36]	Mexico	444 women with low/moderate and high risk of preeclampsia with gestational age between 14 and 32 weeks	L-arginine: 3300 vitamin C: 250; niacin: 25; vitamin B-6: 2; vitamin B-12: 0.0048; folate: 0.2	Vitamin E: 200	Preeclampsia, preterm birth and neonatal death

UK: United Kingdom; USA: United States; mg: milligrams; UI: international units.

**Table 2:** Fetal outcomes meta-analysis and heterogeneity results. Comparison: antioxidants versus placebo.

Outcome	Studies	Population size	Pooled RR	95% CI	*P* value	Heterogeneity tests		
						Chi² *P* value	*I* ^²^	Tau²
Preterm birth	13	21,166	1.03	0.94–1.14	0.51	0.05	43.9%	0.01
Small for gestational age <3rd centile	2	11,634	0.85	0.56–1.30	0.46	0.12	57.9%	0.06
Small for gestational age <5th centile	2	5,320	1.06	0.88–1.28	0.54	0.21	37.6%	0.01
Small for gestational age <10th centile	8	9,672	0.92	0.80–1.05	0.22	0.06	49.2%	0.02
Miscarriage or stillbirth	8	9,209	1.17	0.79–1.74	0.44	0.14	35.6%	0.11
Neonatal death	8	19,135	0.79	0.54–1.17	0.24	0.88	0.0%	0.00
